# Supplementary material for: A mathematical framework for human neutrophil state transitions inferred from single-cell RNA sequence data
Source: Front Immunol. 2025 Oct 24;16:1654015. doi: 10.3389/fimmu.2025.1654015 (PMC12592091; doi:10.3389/fimmu.2025.1654015)
Supplement: Supplementary file 1 [file DataSheet1.pdf]

A mathematical framework for human neutrophil state transitions inferred from single-cell RNA sequence data - Supplementary Material

Gustaf Wigerblad, Jonathan Carruthers, Sumanta Ray, Thomas Finnie, Grant Lythe, Saumyadipta Pyne, Carmen Molina-París, and Mariana J. Kaplan

MCMC sampling

Given cluster fractions derived from the seven healthy samples, MCMC sampling is used to infer parametrizations, $\boldsymbol{\alpha}$, of the Dirichlet distribution, that in turn describes how cluster fractions vary across a general population.

The MCMC sampling is implemented using the emcee Python package with 16 walkers. These walkers are analogous to chains in a Metropolis-Hastings algorithm, but differ in that the proposal distribution for an individual walker depends on the positions of all other walkers. Here, each walker generates $S = 5000$ samples, with the first 500 samples removed as burn-in. Trace plots are provided in Figure S1 for each component of $\boldsymbol{\alpha}$ and give a visual indication that the chains have converged and are well mixed.

Since the samples generated by a walker are not independent, it is important to consider the effective number of independent samples, or effective sample size. The integrated autocorrelation time, τ, represents the number of samples that are needed before the walker forgets where it started, and therefore acts as a measure of the number of steps required to obtain independent samples. With $S = 5000$, the sample size is large enough that estimates of the integrated autocorrelation time are reliable ($S \geq 50\tau$) [1]. The effective sample size for each walker can therefore be estimated as S/τ, giving a total effective sample size of approximately 1200. This suggests there are enough independent samples to accurately estimate the posterior distribution of $\boldsymbol{\alpha}$.

To assess whether the Dirichlet distribution is suitable for describing the cluster fractions, posterior predictive checks are performed. For each MCMC sample, $M = 7$ sets of cluster fractions are sampled from the corresponding Dirichlet distribution and the mean and standard deviation of the cluster fractions are evaluated. Distributions of these means and standard deviations are then compared to their respective values from the healthy sample cluster fractions (Figure S2). This suggests that the fitted Dirichlet distribution overestimates the variability in cluster 0 fractions, allowing for larger values of f_0. Since larger values of f_0 lead to smaller estimates of $\xi_{0,1}$, a statistical model that better captures the variability in f_0 would exclude smaller $\xi_{0,1}$. Such a model reinforces our conclusion that the transition out of cluster 0 occurs on a fast timescale.

Figure S2 also indicates that our statistical model underestimates the variability in cluster 4 fractions. This is likely due to two individuals having a considerably higher fraction of cells in cluster 4 compared to the remaining samples (see Figure 4), increasing the observed standard deviation. Only an increased number of samples would allow us to establish whether these individuals are extreme observations or whether the standard deviation of f_4 truly is underestimated by the model.

Sensitivity of neutrophil clearance rate

The rate at which neutrophils are lost from circulation, μ, is fixed to correspond to a 12-hour half-life. Figure S3 shows how the distribution of each transition rate changes when the half-life, $t_{1/2}$, is assumed to be longer or shorter. Here, the clearance rate is related to the half-life through $\mu = \log(2)/t_{1/2}$. A longer half-life results in a smaller clearance rate, shifting the distributions of the transition rates towards lower values. A shorter half-life shifts the distributions towards higher values. In this case, a $\pm 33\%$ change in the half-life has a limited effect on the transition rates, with the bulk of the distributions covering the same values.

Time-dependent neutrophil influx

Our current model assumes that the influx of neutrophils from bone marrow into circulation occurs with constant rate ϕ. A simple extension to this model is to replace ϕ with a time-dependent function, $\phi(t)$, that

captures the circadian regulation of neutrophils. The system of ODEs is now given by:

$$\frac{dn_0}{dt} = \phi(t) - (\mu + \xi_{0,1})\, n_0 \ , \qquad \frac{dn_1}{dt} = \xi_{0,1} n_0 - (\mu + \xi_{1,2} + \xi_{1,3})\, n_1 \ ,$$
$$\frac{dn_2}{dt} = \xi_{1,2} n_1 - \mu n_2 \ , \qquad \frac{dn_3}{dt} = \xi_{1,3} n_1 - (\mu + \xi_{3,4})\, n_3 \ ,$$
$$\frac{dn_4}{dt} = \xi_{3,4} n_3 - \mu n_4 \ .$$

One possible choice for $\phi(t)$ is a sinusoidal function with a 24-hour period:

$$\phi(t) = A + B \sin\left(\frac{2\pi(t - C)}{24}\right) \ ,$$

where A represents the vertical shift, B is the amplitude and C is the phase shift. Values for A, B and C can be chosen such that the integral of $\phi(t)$ over 24-hours reflects the total number of neutrophils that enter circulation each day. The ratio of the integral over day-time and night-time hours also indicates the strength of the circadian rhythm.

Here, we set $A = 2.08 \times 10^9$ cells, $B = 10^9$ to give a total daily influx of 5×10^{10} cells. For simplicity, we set $C = 0\ h$. The transition rates are $\xi_{0,1} = 4.84\ h^{-1}$, $\xi_{1,2} = 0.017\ h^{-1}$, $\xi_{1,3} = 0.10\ h^{-1}$ and $\xi_{3,4} = 0.026\ h^{-1}$, and correspond to a single draw from the MCMC sample.

References

[1] Daniel Foreman-Mackey. Autocorrelation analysis & convergence. Accessed 06/08/2025. Available at `https://emcee.readthedocs.io/en/stable/tutorials/autocorr`.

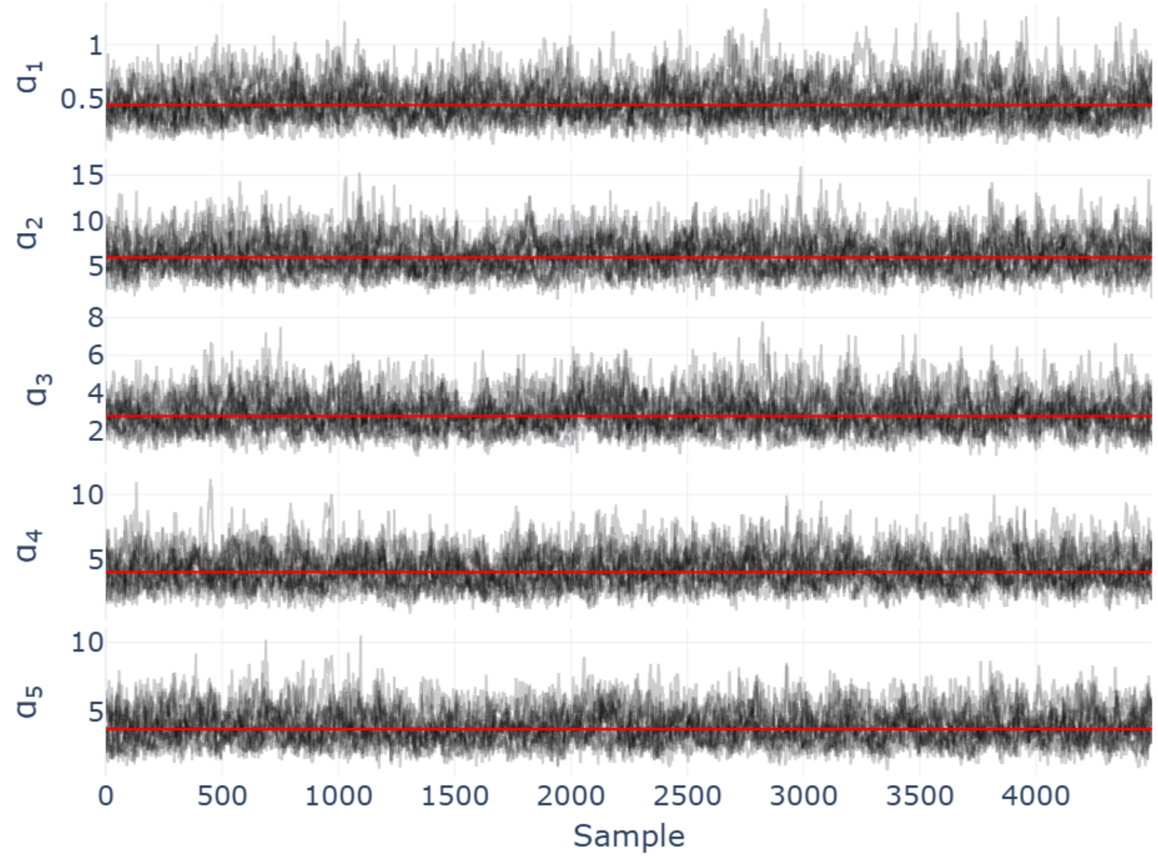

Figure S1: Trace plots of the MCMC samples for each component of the vector $\boldsymbol{\alpha} = (\alpha_1, ..., \alpha_5)$ that is used to parametrize the Dirichlet distribution. Red lines indicate posterior median estimates.

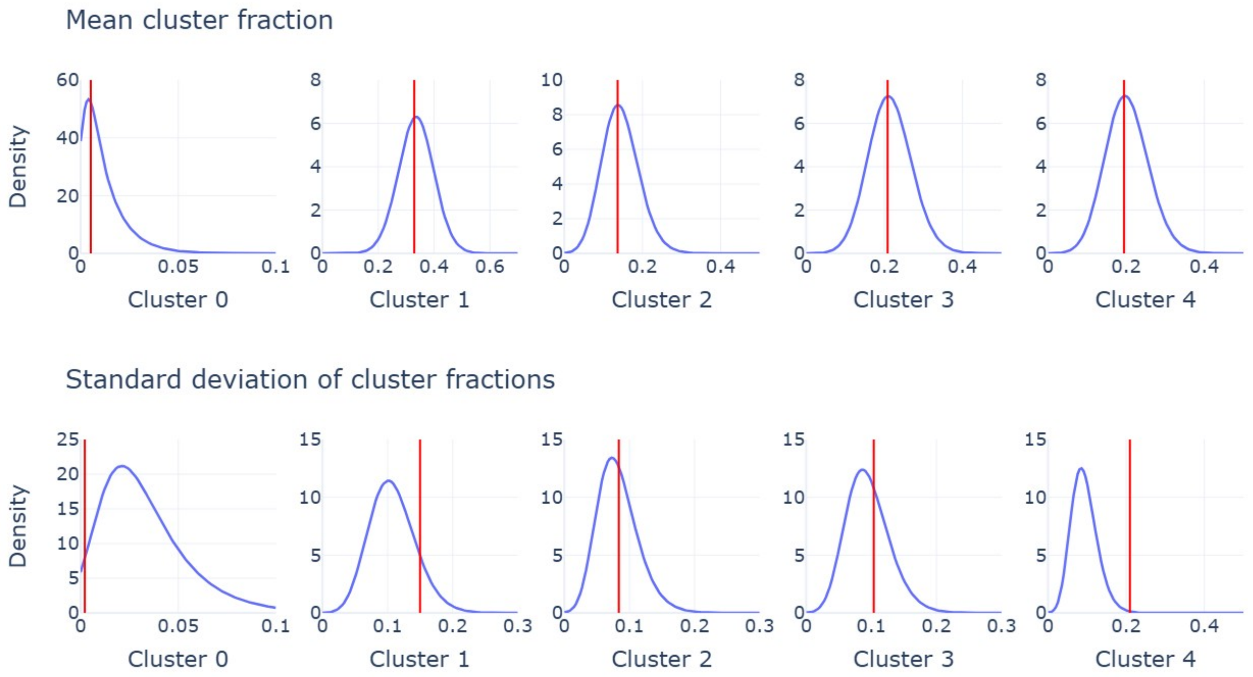

Figure S2: Posterior predictive checks for the mean and standard deviation of cluster fractions. Vertical red lines correspond to the mean and standard deviation of cluster fractions from healthy samples.

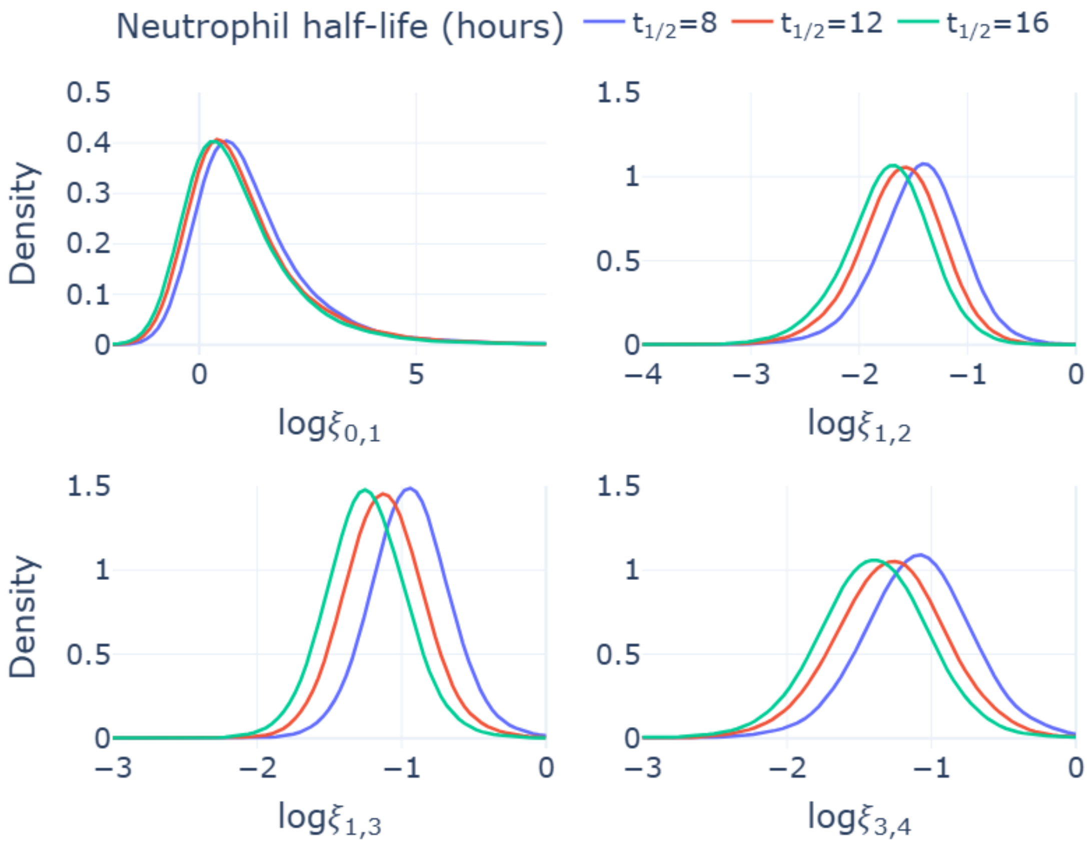

Figure S3: An indication of how the densities of the transition rates, $\xi_{i,j}$, change under different neutrophil half-lives. The half-life and loss rate are related by $\mu = \log(2)/t_{1/2}$.
